# Comparative Transcriptomic Profiling Identifies Region- and Phenotypic-Background-Associated Transcriptional Programs Linked to Coat-Color Variation in Junken Meat Sheep

**DOI:** 10.3390/biology15171457

**Published:** 2026-08-26

**Authors:** Binpeng Xi, Sanchuan Zhao, Qian Yu, Wenzhe Zhang, Yan Chen, Zhipeng Wang, Hua Yang, Jianbin Liu

**Affiliations:** 1 State Key Laboratory of Sheep Genetic Improvement and Healthy Production, Xinjiang Academy of Agricultural and Reclamation Science, Shihezi 832000, China; w5562080w@163.com (B.X.); lth112502@163.com (S.Z.); sugeryq@163.com (Q.Y.); zwz561565@gmail.com (W.Z.); 15739335607@163.com (Y.C.); 2 Key Laboratory of Animal Genetics and Breeding on the Tibetan Plateau, Ministry of Agriculture and Rural Affairs, Lanzhou Institute of Husbandry and Pharmaceutical Sciences, Chinese Academy of Agricultural Sciences, Lanzhou 730050, China; 3 Sheep Breeding Engineering Technology Research Center, Chinese Academy of Agricultural Sciences, Lanzhou 730050, China; 4 College of Animal Science and Technology, Northeast Agricultural University, Harbin 150030, China; wangzhipeng@neau.edu.cn

**Keywords:** Junken meat sheep, coat-color variation, skin transcriptome, melanogenesis, follicular microenvironment

## Abstract

Junken meat sheep display distinct coat-color phenotypes, including a white body with a black head and an entirely black coat at birth, but the transcriptional basis of variation across skin regions and phenotypic backgrounds remains unclear. We performed bulk RNA sequencing (RNA-seq) on nine skin samples from six 1-month-old lambs: black-haired head skin (BT), white-haired dorsal trunk skin (WT), and black-haired dorsal trunk skin from lambs born entirely black (B), with three biological replicates per group. The paired BT–WT comparison characterized within-individual regional differences, whereas B–WT represented a composite phenotypic-background comparison. Cross-comparison integration identified 2056 region-associated genes, 8027 phenotypic-background-associated genes, and 5615 shared genes. Region-associated genes were enriched in mitochondrial and lipid-metabolic processes, phenotypic-background-associated genes in cell communication and adhesion, and shared genes in epidermal keratinization, cytoskeletal organization, and extracellular matrix–integrin-related processes. Network analysis identified a pigmentation-effector subnetwork containing *TYR*, *TYRP1*, *PMEL*, *OCA2*, *SLC45A2* and *MYO5A*. Signature scoring suggested that some contractile signals may reflect tissue-composition differences. Reverse transcription quantitative PCR broadly supported the RNA-seq expression trends. These findings provide a multilayered transcriptional framework for subsequent functional validation of coat-color variation in Junken meat sheep.

## Simple Summary

Junken meat sheep show distinctive coat-color patterns, including animals with a black head and white body and animals that are entirely black at birth. However, it remains unclear why gene activity differs between black- and white-haired skin and between these coat-color types. In this study, we compared gene activity in black-haired head skin, white-haired trunk skin, and black-haired trunk skin from one-month-old Junken meat sheep. The results showed that coat-color differences were associated not only with genes directly involved in producing, storing, and transporting pigment, but also with genes related to skin structure, cell attachment, the local environment around hair follicles, energy use, and protection against damaging oxygen-related molecules. Some signals linked to muscle and contraction may have reflected differences in the depth or composition of the collected skin rather than direct control of pigmentation. Overall, the study provides a broader explanation of coat-color variation in Junken meat sheep and identifies several groups of candidate genes for future testing. These findings may support coat-color breeding, improve the description and conservation of sheep genetic resources, and guide further studies of skin and hair-follicle biology.

## 1. Introduction

Coat color is an important phenotypic trait shaped by long-term domestication and artificial selection in sheep, and population-specific coloration and patterning also provide important criteria for breed identification and the characterization of genetic resources [1]. Two coat-color lines are recognized within the Junken meat sheep breeding population. Lambs of one line are entirely black at birth, after which the trunk progressively turns white during growth while the head remains black; lambs of the other line exhibit a black head and white body at birth, with a relatively stable spatial distribution of coat color thereafter [2]. Genetically, coat color in sheep is jointly regulated by multiple pigmentation-associated loci, among which *ASIP* and *MC1R* are the most well-established major genes. Allelic variation at these loci can alter melanocortin signaling and the balance between eumelanin and pheomelanin synthesis, thereby generating diverse coat colors and pigmentation patterns [3,4,5,6]. However, genetic variation must be translated into a visible coat phenotype through a series of cellular processes, including melanocyte differentiation, melanin synthesis, melanosome maturation, and pigment transfer to the hair shaft [7,8]. The transcriptional states of the skin and hair follicle therefore represent an important regulatory layer linking genetic background to coat-color phenotype; however, their variation across coat-color lines and body regions in Junken meat sheep has yet to be systematically characterized.

Hair follicle pigmentation is not driven by melanocytes alone, but is jointly regulated by the melanocyte lineage, follicular epithelium, and surrounding mesenchyme. During anagen, mature melanocytes synthesize melanin and transfer melanosomes to adjacent hair matrix keratinocytes, where pigment is incorporated into the keratinizing hair shaft to produce visible coat color [9]. Melanocyte stem cells and epithelial hair follicle stem cells form an interdependent niche, and their coordinated activation supports melanocyte replenishment and pigmented hair regeneration [10,11]. The dermal papilla may also influence pigmentation by modulating the hair cycle, hair matrix cell behavior, and the local paracrine environment. Thus, coat color depends not only on melanogenic activity, but also on pigment-cell localization, follicular-cycle status, and local intercellular communication. Single-cell transcriptomic studies further show that sheep hair follicles and surrounding skin comprise diverse cell populations with distinct transcriptional states involved in follicle morphogenesis, hair shaft formation, extracellular matrix remodeling, and pigment deposition [12].

Previous transcriptomic and multi-omics studies in sheep and goats support this view. Comparisons among coat-color breeds, black- and white-haired skin regions, and developmental stages indicate that coat-color variation is associated not only with melanogenesis and its upstream signaling networks, but also with hair follicle development, cell adhesion, extracellular matrix remodeling, and skin structural maintenance [13,14,15,16,17,18]. Collectively, histological and multi-omics evidence suggests that visible coat color reflects the combined effects of genetic background, melanocyte function, and the local tissue environment, rather than the expression of a few canonical pigmentation genes alone.

Accordingly, this study investigated coat-color-associated transcriptional variation across skin regions and phenotypic backgrounds in 1-month-old Junken meat sheep. Two biologically distinct transcriptomic comparisons were established. BT and WT samples were collected from the black-haired head region and white-haired dorsal trunk region, respectively, of the same white-bodied, black-headed individuals to characterize within-individual regional expression differences. The B–WT comparison was used to assess composite transcriptional differences between black-haired dorsal trunk skin from born-black sheep and white-haired dorsal trunk skin from white-bodied, black-headed sheep. By integrating differential expression analysis, cross-comparison gene stratification, functional enrichment, network-based prioritization, and sample-level signature scoring, we aimed to identify region-associated, phenotypic-background-associated, and shared transcriptional features. These findings provide transcriptomic evidence for understanding developmental coat-color variation in Junken meat sheep and offer a basis for further studies of sheep coat-color genetics and the utilization of local genetic resources.

## 2. Materials and Methods

### 2.1. Ethics Statement

The animal procedures used in this study complied with applicable standards for laboratory-animal welfare. Ethical authorization was granted by the Institutional Animal Care and Use Committee of the Xinjiang Academy of Agricultural and Reclamation Sciences, Shihezi, China, under protocol XJNKKXY-2020-34 on 30 December 2020. Animal manipulation and tissue sampling were carried out with measures designed to limit pain, distress, and unnecessary discomfort.

### 2.2. Experimental Animals and Sample Collection

Six one-month-old Junken meat sheep of the male were obtained from the Sheep Breeding Farm of the Xinjiang Academy of Agricultural and Reclamation Sciences and maintained under uniform husbandry conditions. Nine skin samples were classified as black-haired head skin (BT), black-haired dorsal trunk skin (B), or white-haired dorsal trunk skin (WT), with three biological replicates per group. Paired BT and WT samples were collected from the same three white-bodied, black-headed sheep to assess within-individual regional coat-color-associated differences while reducing between-animal variation, whereas B samples were obtained from three additional sheep with an entirely black coat at birth and compared with WT samples from the same dorsal trunk region to evaluate transcriptional variation across different coat-color phenotypic backgrounds. No biological pairing was established between B and WT animals; the identifiers B1–B3 and WT1–WT3 denote replicate labels only and do not indicate one-to-one animal correspondence. Skin biopsies were performed on live animals under veterinary supervision after fleece removal, disinfection, and local anesthesia with 1% lidocaine hydrochloride (3 mg/kg body weight; maximum total dose, 6 mg/kg). Full-thickness skin was collected aseptically, wounds were sutured after hemostasis, and meloxicam (0.5 mg/kg) was administered for postoperative analgesia. Samples were snap-frozen in liquid nitrogen and stored at −80 °C until RNA extraction. Animal and sampling details are provided in Supplementary Table S1.

### 2.3. RNA Extraction

Total RNA was isolated from B, BT, and WT skin tissues using TRIzol reagent in combination with the RNeasy RNA Purification Kit (Servicebio, Wuhan, China), following the manufacturer’s protocols. RNA concentration and purity were determined using a NanoDrop™ One spectrophotometer (Thermo Fisher Scientific, Waltham, MA, USA), and RNA integrity was evaluated by electrophoresis on a 1% agarose gel.

### 2.4. Library Construction, Sequencing, and Bioinformatic Processing

Library preparation and sequencing were performed by Novogene Co., Ltd. (Beijing, China). Approximately 5 μg of total RNA from each sample was used as the input material. Following mRNA enrichment and random fragmentation, first- and second-strand cDNA synthesis was performed. The resulting cDNA fragments underwent end repair, 3′-end adenylation, adapter ligation, size selection, and PCR amplification to generate strand-specific libraries using the NEBNext^®^ Ultra™ Directional RNA Library Prep Kit (New England Biolabs, Ipswich, MA, USA). Library concentration, fragment-size distribution, and effective concentration were assessed using a Qubit 2.0 Fluorometer (Thermo Fisher Scientific, Waltham, MA, USA), an Agilent 2100 Bioanalyzer (Agilent Technologies, Santa Clara, CA, USA), and quantitative PCR, respectively. Qualified indexed libraries were clustered and sequenced on an Illumina NovaSeq 6000 platform (Illumina, San Diego, CA, USA) to generate 150-bp paired-end reads. Raw imaging data were subjected to base calling with Illumina CASAVA and converted into FASTQ files. Sequence quality control was performed using fastp v0.23.4, and reads containing adapter contamination, excessive ambiguous nucleotides, or low-quality bases were removed. The resulting clean reads were aligned against the ovine reference genome Oar_rambouillet_v1.0 using HISAT2 for subsequent gene-expression quantification and differential-expression analysis. The raw mRNA-seq data are available in the NCBI Sequence Read Archive under Bio Project accession PRJNA1492038.

### 2.5. Functional Enrichment and Network Analysis of Differentially Expressed Genes

Genes with an absolute |log2 fold change| ≥ 1 and adjusted *p* < 0.05 were defined as differentially expressed genes (DEGs). Gene Ontology (GO) and Kyoto Encyclopedia of Genes and Genomes (KEGG) enrichment analyses were subsequently conducted using the cluster Profiler package. GO terms and KEGG pathways with an adjusted *p* value below 0.05 were considered significantly enriched. Protein–protein interaction networks of selected DEGs were generated using the STRING database (accessed on 12 June 2026) and visualized in Cytoscape v3.7.1. Candidate genes were prioritized according to network connectivity, subnetwork membership, and functional relevance.

### 2.6. Marker-Based Signature Construction and Sample-Level Scoring

To further characterize sample-level transcriptional patterns, six marker-based signatures were constructed for melanocyte/melanosome, keratinocyte/epidermal, fibroblast/extracellular matrix (ECM), immune/inflammatory, lipid/redox metabolism, and contractile/muscle-like programs. Marker genes were curated from genes represented in the transcriptomic dataset according to their established cellular or functional associations, and the complete gene sets are provided in Supplementary Table S4. Expression values were transformed as log2(FPKM + 1), standardized across the nine samples using gene-wise Z-scores, and the score for each signature was calculated as the mean Z-score of its detected marker genes within each sample. Of the 69 predefined markers, KRT14 was not detected and was excluded, leaving 68 genes for the final scoring. The resulting scores were used to compare the relative representation and heterogeneity of these transcriptional programs across B, BT, and WT samples and were interpreted as relative bulk-transcriptomic signatures rather than direct estimates of cell-type abundance or tissue composition.

### 2.7. Reverse Transcription Quantitative PCR Validation

Twelve differentially expressed genes were selected for validation by reverse transcription quantitative PCR (RT-qPCR). Gene-specific primers were designed using Primer Premier 5.0 and synthesized by Sangon Biotech (Shanghai, China); the primer sequences are provided in Supplementary Table S2. Total RNA was reverse-transcribed into complementary DNA using the Revert Aid First Strand cDNA Synthesis Kit (Thermo Fisher Scientific, Waltham, MA, USA) according to the manufacturer’s instructions. RT-qPCR was performed using the Quanti Nova SYBR Green PCR Kit (QIAGEN, Shanghai, China). Ovine GAPDH was used as the reference gene, and relative transcript abundance was calculated using the 2^−ΔΔCt^ method. Each reaction was performed in triplicate.

### 2.8. Statistical Analysis

Statistical analyses were performed using GraphPad Prism 8.0. Data are presented as the mean ± standard error of the mean (SEM). Paired comparisons between BT and WT samples were conducted using a paired Student’s *t*-test, whereas comparisons between independent groups were performed using an unpaired Student’s *t*-test. When all three groups were analyzed simultaneously, one-way analysis of variance (ANOVA) followed by Bonferroni’s multiple-comparison test was applied. Differences were considered statistically significant at *p* < 0.05.

## 3. Results

### 3.1. Sampling Design and Analytical Framework for Resolving Coat-Colour-Associated Transcriptional Variation

**Figure 1:**
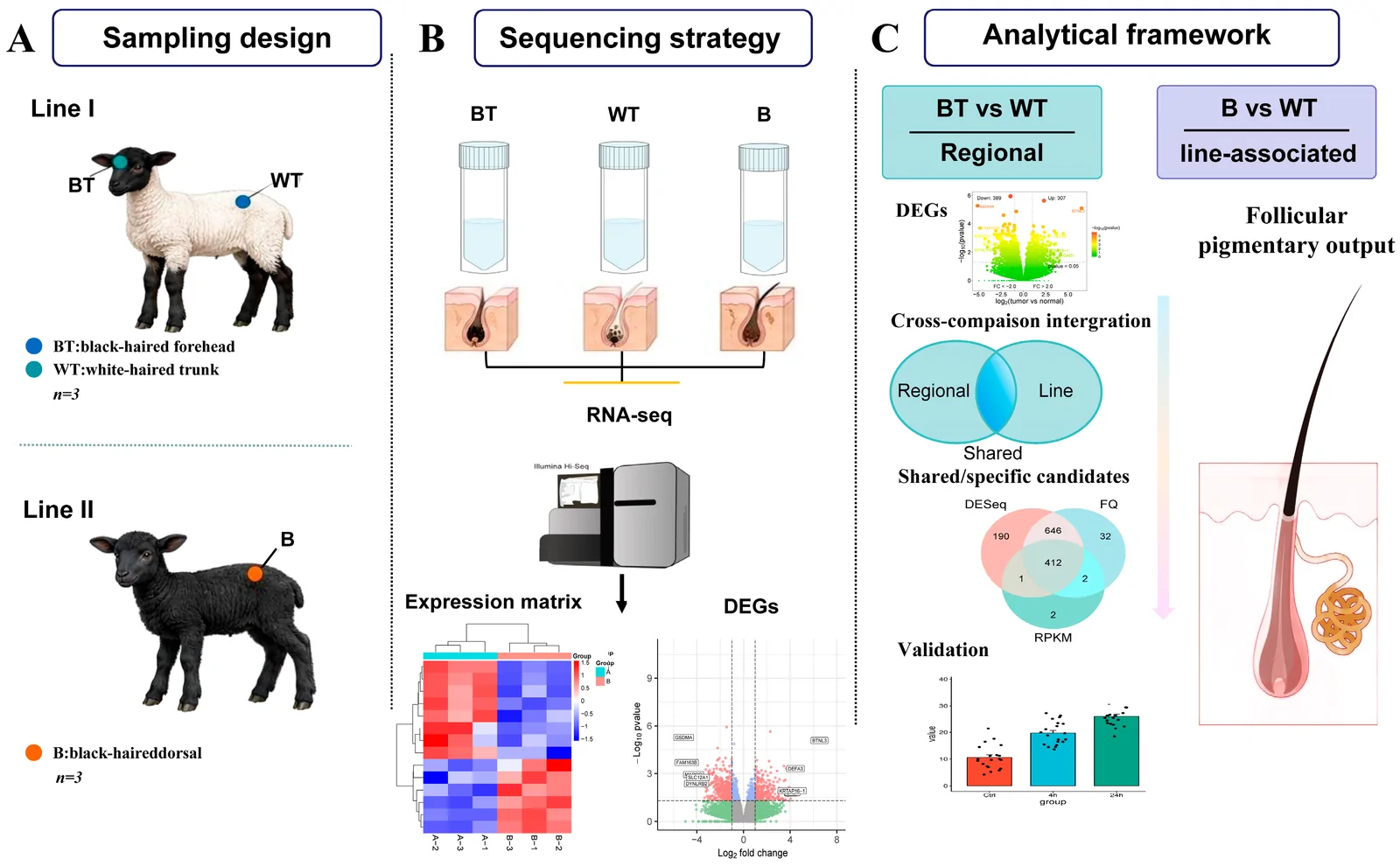
Sampling design and analytical framework for transcriptomic profiling of coat-color-associated skin variation in Junken meat sheep. (**A**) Sampling design for BT, WT, and B skin samples. (**B**) RNA-seq workflow for expression quantification and differential-expression analysis. (**C**) Cross-comparison integration of the BT–WT and B–WT DEG sets, followed by stratification into region-associated, line-background-associated, and shared transcriptional mod-ules and subsequent downstream analyses.

To characterize coat-color-associated transcriptional variation in Junken meat sheep skin, BT, WT, and B samples were analyzed, with three biological replicates per group. Paired BT and WT samples were collected from the black-haired head and white-haired dorsal trunk regions of the same white-bodied, black-headed sheep for within-individual regional comparison, whereas B samples were obtained from the black-haired dorsal skin of born-black sheep and compared with WT to assess line-background-associated variation (Figure 1A). The BT, WT, and B groups generated 42.38 ± 2.14, 44.29 ± 0.52, and 45.29 ± 2.49 million raw reads, respectively, of which 41.09 ± 2.63, 43.11 ± 0.71, and 44.30 ± 2.42 million clean reads were retained after filtering. Corresponding clean data yields were 6.17 ± 0.40, 6.47 ± 0.11, and 6.65 ± 0.36 Gb, with sequencing error rates of 0.02 ± 0.00%, 0.02 ± 0.01%, and 0.03 ± 0.00%, respectively (Supplementary Table S3). These metrics supported subsequent expression quantification and downstream analyses. Two complementary comparison frameworks were therefore established: BT–WT for region-associated differences and B–WT for line-background-associated differences (Figure 1B). The analytical workflow included differential-expression screening, cross-comparison integration, gene stratification, functional enrichment, GSEA, network-based prioritization, and sample-level signature scoring (Figure 1C).

### 3.2. Differential-Expression Screening Reveals Broad but Heterogeneous Transcriptional Differences Among Coat-Colour-Associated Skin Samples

**Figure 2:**
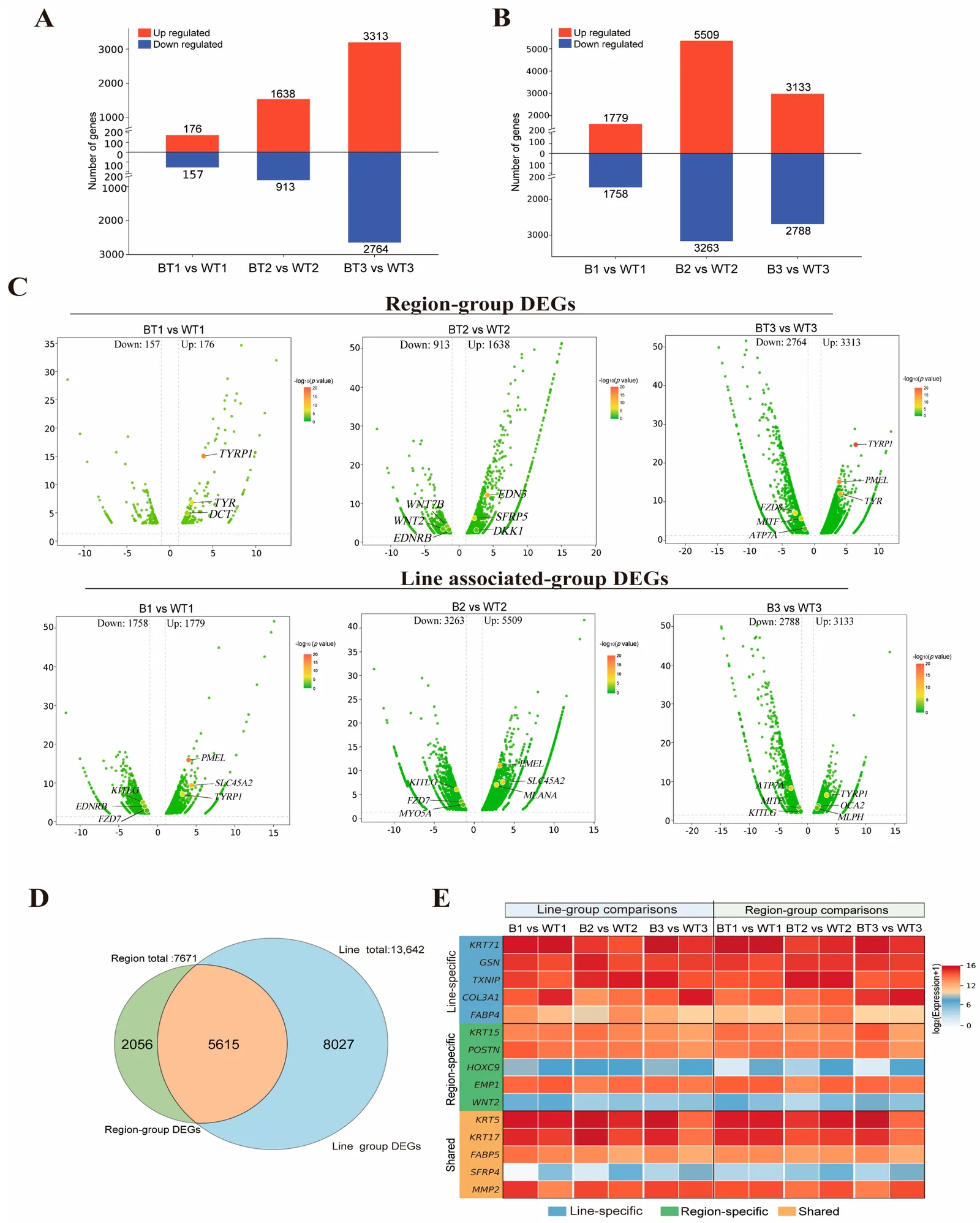
Differential-expression landscapes across regional and line-background-associated skin comparisons. (**A**,**B**) Numbers of upregulated and downregulated DEGs in the BT–WT and B–WT comparisons, respectively. (**C**) Volcano plots of DEGs and representative coat-color-associated genes. (**D**) Overlap between region-associated and line-background-associated DEG sets. (**E**) Expression heatmap of representative genes from the three DEG categories. For B–WT, numerical identifiers denote replicate-level contrast labels and not biologically matched animals.

Differential-expression analysis identified extensive transcriptional variation in both comparison frameworks, although the magnitude and consistency of the changes differed among samples. In the region-associated comparisons, BT1–WT1, BT2–WT2, and BT3–WT3 contained 176/157, 1638/913, and 3313/2764 upregulated/downregulated genes, respectively (Figure 2A), indicating substantial inter-individual heterogeneity in the transcriptional differences between black- and white-haired skin regions within the same coat-color phenotype. The line-background-associated contrasts generally yielded larger DEG sets, with 1779/1758, 5509/3263, and 3133/2788 upregulated/downregulated genes detected in B1–WT1, B2–WT2, and B3–WT3, respectively (Figure 2B).

Volcano plots further showed that genes associated with melanogenesis and the follicular microenvironment were distributed across both comparison frameworks (Figure 2C). In the region-associated contrasts, canonical pigmentation effector genes, including *TYRP1*, *TYR* and *DCT*, were differentially expressed in selected sample comparisons, together with Wnt/EDN-related genes such as *WNT2*, *WNT7B*, *EDNRB*, *EDN3*, *SFRP5* and *DKK1*. In the line-background-associated contrasts, differential expression was observed for genes involved in melanosome formation, maturation, and transport, including *PMEL*, *SLC45A2*, *TYRP1*, *MLANA* and *OCA2*, as well as genes associated with melanocyte development or melanosome trafficking, including *KITLG*, *EDNRB*, *FZD7* and *MYO5A*. Cross-comparison integration identified 7671 region-associated and 13,642 line-background-associated DEGs. Of these, 2056 were unique to the region-associated set, 8027 were unique to the line-background-associated set, and 5615 were shared between the two sets (Figure 2D). Heatmaps of representative genes revealed distinguishable expression patterns among the three DEG categories across samples and comparison frameworks, providing a basis for subsequent module-level analyses (Figure 2E).

### 3.3. DEG Stratification Separates Comparison-Specific and Shared Transcriptional Modules

**Figure 3:**
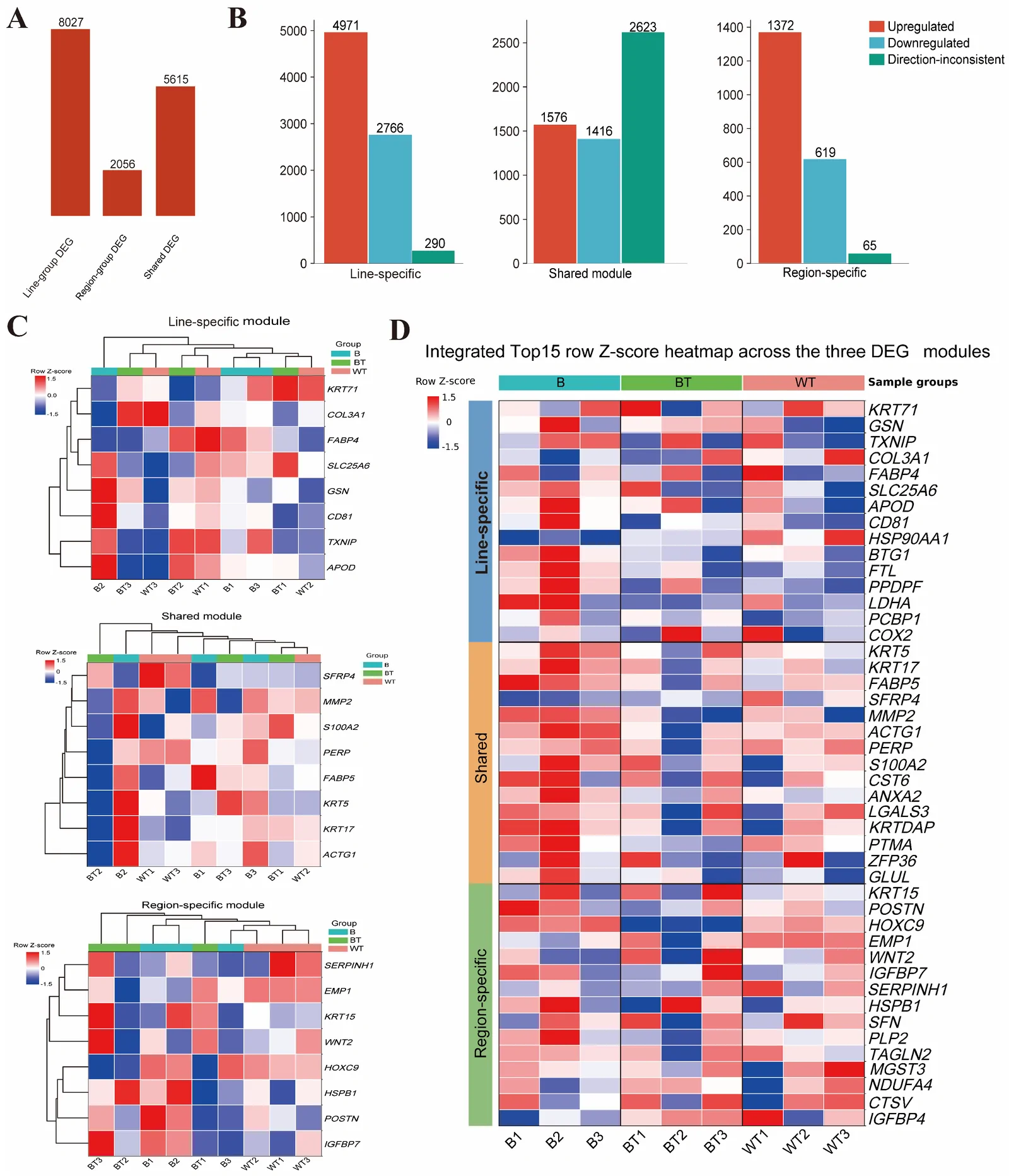
Stratification of DEGs into line-background-associated, region-associated, and shared transcrip-tional modules. (**A**) Numbers of DEGs assigned to the three modules. Orange, cyan-green, and magenta indicate the line-background-associated, region-associated, and shared modules, respec-tively. (**B**) Expression-direction composition of each module, including upregulated, downregu-lated, and direction-inconsistent genes. (**C**) Row Z-score heatmaps of representative genes from the three modules. (**D**) Integrated row Z-score heatmap of the top 15 genes from each module across B, BT, and WT samples.

Based on their distribution across the two comparison frameworks, DEGs were classified into line-background-associated, region-associated, and shared categories, comprising 8027, 2056, and 5615 genes, respectively (Figure 3A). The B–WT comparison contributed a larger number of comparison-specific genes, whereas the shared category comprised genes detected in both comparison frameworks. Analysis of expression direction revealed distinct levels of consistency among the three categories. The line-background-associated category contained 4971 upregulated, 2766 downregulated, and 290 direction-inconsistent genes, accounting for 61.9%, 34.5%, and 3.6% of the category, respectively. The region-associated category contained 1372 upregulated, 619 downregulated, and 65 direction-inconsistent genes, representing 66.7%, 30.1%, and 3.2%, respectively. In contrast, the shared category comprised 1576 upregulated, 1416 downregulated, and 2623 direction-inconsistent genes, corresponding to 28.1%, 25.2%, and 46.7%, respectively (Figure 3B). Thus, the line-background-associated and region-associated categories showed relatively consistent directional profiles, whereas the shared category contained a larger proportion of genes whose expression direction depended on the comparison context.

Heatmaps of representative genes supported this classification. Within the line-background-associated category, *KRT71*, *GSN*, *TXNIP*, *COL3A1*, *FABP4*, *SLC25A6*, *APOD* and *CD81* displayed distinct expression patterns across B, BT, and WT samples. Region-associated genes, including *KRT15*, *POSTN*, *HOXC9*, *EMP1*, *WNT2*, *IGFBP7*, *SERPINH1* and *HSPB1*, showed region-dependent expression differences, whereas *KRT5*, *KRT17*, *FABP5*, *SFRP4*, *MMP2*, *ACTG1*, *PERP* and *S100A2* exhibited distinguishable sample-dependent patterns within the shared category (Figure 3C). Row Z-score heatmaps of the top 15 representative genes from each category further highlighted differences in expression direction and sample-level patterns among the three DEG classes (Figure 3D).

### 3.4. Functional Enrichment and GSEA Reveal Distinct Biological Features of Line-Specific, Region-Specific and Shared Gene Modules

**Figure 4:**
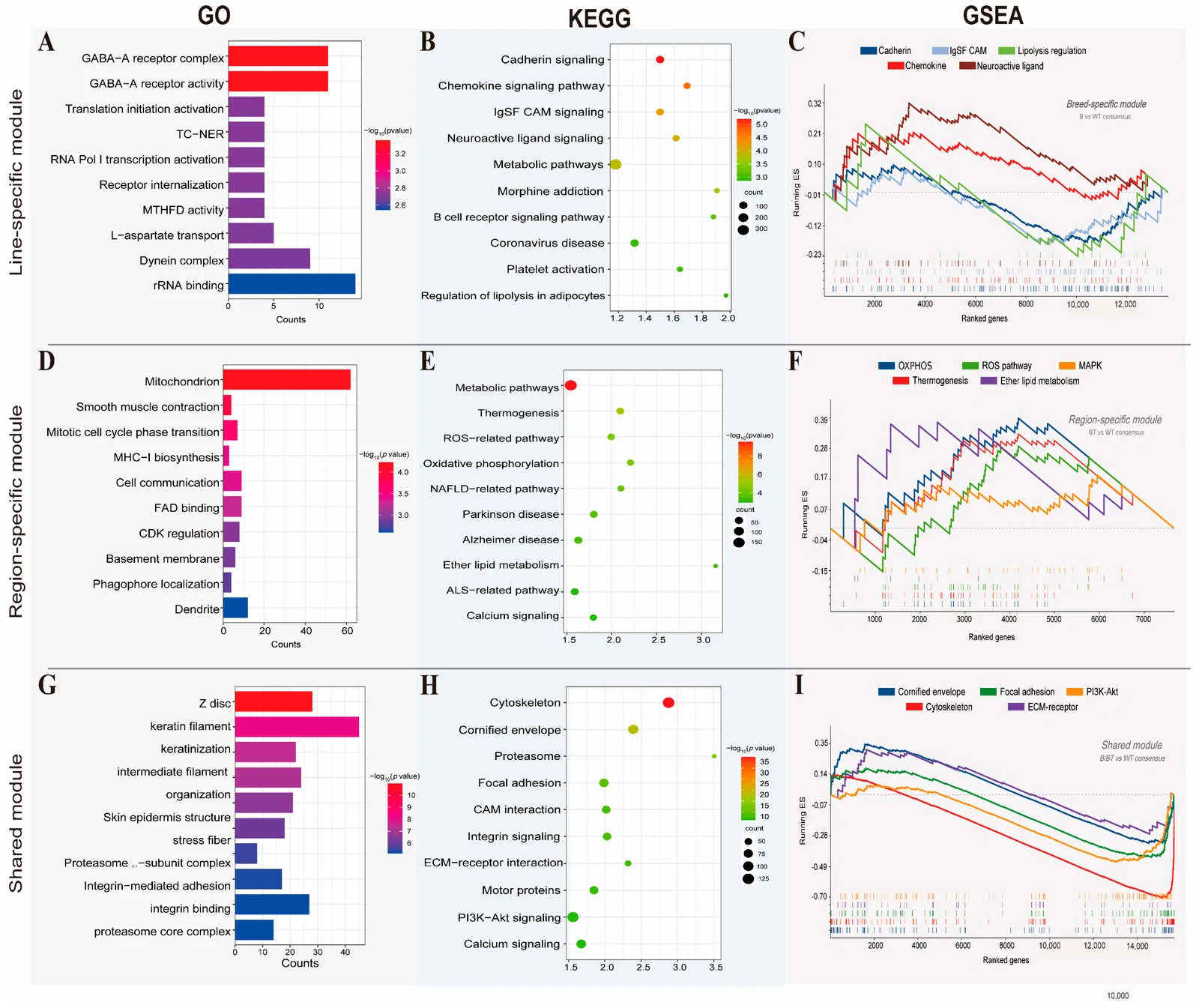
Functional enrichment and GSEA analyses of module-specific genes associated with coat-color divergence. GO, KEGG and GSEA analyses were performed for the Line-specific, shared and region-specific gene modules. GO, KEGG, and GSEA results are shown for the line-background-associated (**A**–**C**), region-associated (**D**–**F**), and shared (**G**–**I**) modules.

The three DEG categories exhibited distinct functional enrichment profiles rather than converging on a single melanogenesis pathway. To compare their major biological features, Gene Ontology (GO), Kyoto Encyclopedia of Genes and Genomes (KEGG), and representative gene set enrichment analyses were performed separately for the line-background-associated, region-associated, and shared gene sets (Figure 4).

The line-background-associated category was primarily related to signal perception, cell communication, cell adhesion, and lipid metabolic regulation. Prominent GO terms included GABA-A receptor complex, GABA-A receptor activity, receptor internalization, activation of RNA polymerase I transcription, dynein complex, and rRNA binding (Figure 4A). KEGG analysis further implicated cadherin signaling, chemokine signaling, immunoglobulin superfamily cell adhesion molecule signaling, neuroactive ligand signaling, metabolic pathways, and regulation of lipolysis in adipocytes (Figure 4B). Consistently, GSEA supported enrichment trends for pathways associated with cell adhesion, chemokine signaling, neuroactive ligand signaling, and lipolytic regulation in the line-background-associated comparison (Figure 4C). The region-associated category showed more pronounced mitochondrial, energetic, and redox-related features. Mitochondrion was the most prominent GO term, accompanied by enrichment of FAD binding, basement membrane, cyclin-dependent kinase regulation, phagophore localization, cell communication, and mitotic cell-cycle phase transition (Figure 4D). KEGG analysis indicated enrichment of metabolic pathways, thermogenesis, reactive oxygen species-related processes, oxidative phosphorylation, ether lipid metabolism, and calcium signaling (Figure 4E). GSEA yielded concordant enrichment trends for oxidative phosphorylation, thermogenesis, reactive oxygen species-related pathways, ether lipid metabolism, and MAPK signaling (Figure 4F). The shared category was more strongly associated with epidermal structure, keratinization, cytoskeletal organization, adhesion, and extracellular matrix-related processes. GO analysis revealed significant enrichment of keratin filament, keratinization, intermediate filament organization, epidermal structure, stress fiber, integrin-mediated adhesion, integrin binding, and proteasome-related terms (Figure 4G). KEGG analysis further identified cytoskeletal organization, cornified envelope formation, focal adhesion, cell adhesion molecule interactions, integrin signaling, ECM–receptor interaction, PI3K–Akt signaling, and calcium signaling (Figure 4H). GSEA supported enrichment of cornified-envelope formation, cytoskeletal organization, adhesion, ECM–receptor interaction, and PI3K–Akt signaling within the shared category (Figure 4I).

Overall, line-background-associated genes were mainly linked to cell communication, adhesion, and lipolytic regulation; region-associated genes were enriched in mitochondrial function, energy metabolism, and redox processes; and shared genes were predominantly associated with epidermal keratinization, cytoskeletal organization, and extracellular matrix remodeling. These findings indicate that transcriptional differences among differently colored skin regions in Junken meat sheep extend beyond canonical melanogenesis and involve multiple layers of metabolic state, tissue architecture, and the local microenvironment.

### 3.5. Network Prioritization Identifies Module-Specific Candidate Genes and a Shared Melanogenesis-Associated Subnetwork

**Figure 5:**
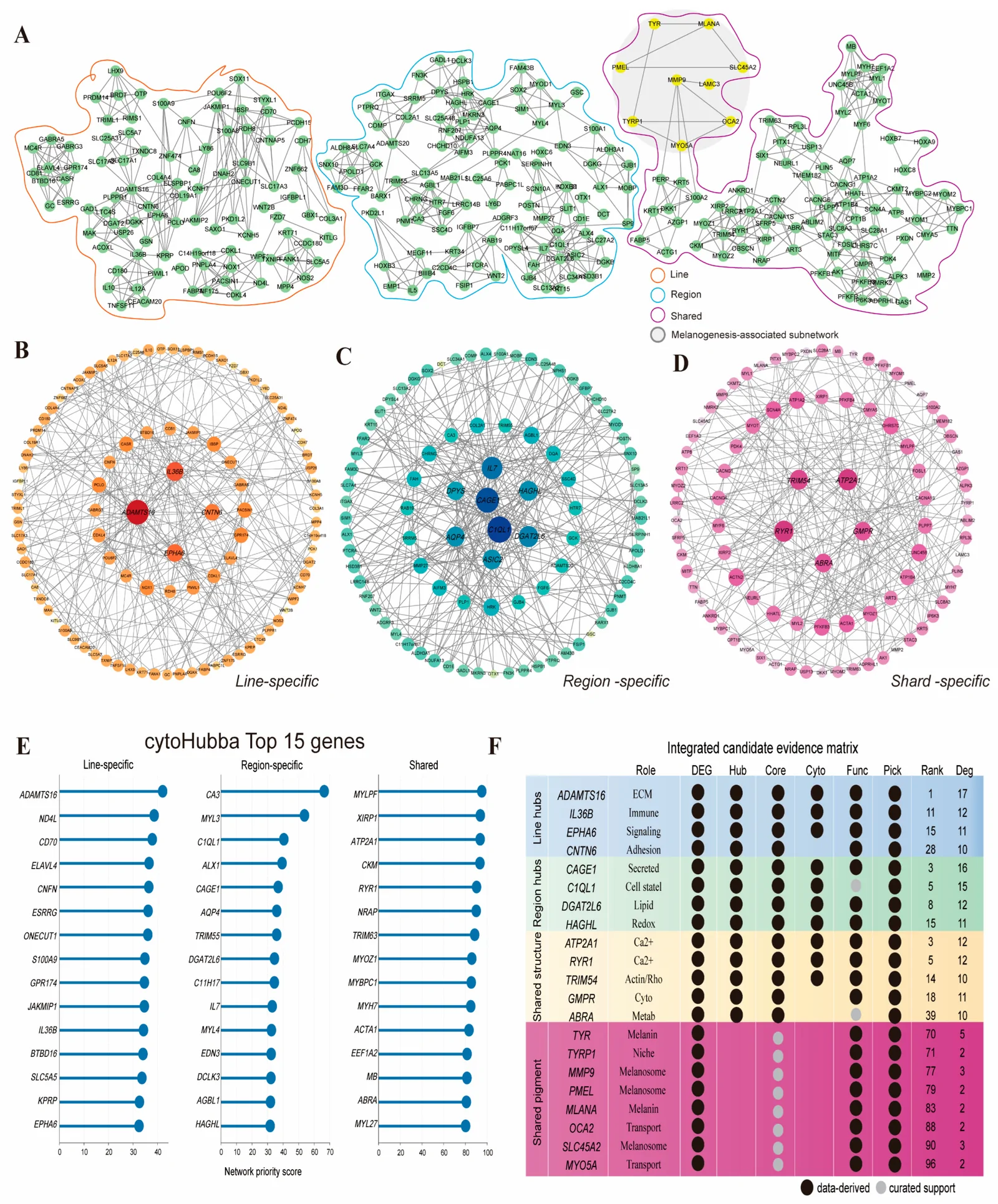
Network-based prioritization of module-specific hub genes and a shared melanogene-sis-associated subnetwork in Junken meat sheep skin. (**A**) Network overview of the three DEG modules. Orange, cyan-green, and magenta delineate the line-background-associated, re-gion-associated, and shared modules, respectively, whereas yellow highlights the melanogene-sis-associated subnetwork within the shared module. (**B**–**D**) Core subnetworks of the line-background-associated, region-associated, and shared modules, respectively. (**E**) Top 15 network-ranked genes in each module. (**F**) Integrated evidence matrix for candidate-gene priori-tization.

To prioritize key candidate genes, protein–protein interaction networks were constructed separately for the line-background-associated, region-associated, and shared gene sets. Candidate genes were then ranked by integrating core-subnetwork membership, cytoHubba scores, and an evidence matrix (Figure 5).

The three networks displayed distinct connectivity patterns, enabling the identification of highly connected nodes and functionally coherent subnetworks (Figure 5A). In the line-background-associated category, the core network included *ADAMTS16*, *IL36B*, *EPHA6* and *CNTN6*, with *ADAMTS16* ranking among the top cytoHubba candidates (Figure 5B,E). Based on functional annotation, these genes were mainly associated with extracellular matrix remodeling, inflammatory signaling, and cell adhesion and were therefore retained as candidate markers of the line-background-associated skin microenvironment. Within the region-associated category, *CAGE1*, *C1QL1*, *DGAT2L6* and *HAGHL* exhibited relatively high network centrality, whereas *CA3*, *MYL3*, *ALX1*, *AQP4*, *TRIM55* and *IL7* also ranked highly in the cytoHubba analysis (Figure 5C,E). Functionally, *C1QL1* is associated with extracellular secretory signaling, *DGAT2L6* with lipid metabolism or remodeling, *HAGHL* with glutathione-related metabolism, and *CA3* and *AQP4* with local acid–base buffering and water–ion homeostasis, respectively. On the basis of network position and functional relevance, *C1QL1*, *DGAT2L6*, *HAGHL* and *CA3* were prioritized as representative region-associated candidates, collectively reflecting metabolic, redox, and local homeostatic features. The core network of the shared category was dominated by genes related to Ca^2+^ homeostasis and the contractile cytoskeleton, including *MYLPF*, *XIRP1*, *ATP2A1*, *CKM*, *RYR1* and *MYH7*, which ranked highly in the cytoHubba analysis (Figure 5D,E). *ATP2A1*, *RYR1*, *TRIM54*, *GMPR* and *ABRA* were retained as representative nodes of this category.

In addition to highly central nodes, a functional subnetwork composed primarily of canonical pigmentation effector genes was identified within the shared category (Figure 5A). This subnetwork included *TYR*, *TYRP1*, *PMEL*, *MLANA*, *OCA2*, *SLC45A2* and *MYO5A*, spanning melanin synthesis, melanosome formation and maturation, and intracellular transport, and was connected to the matrix-remodeling node *MMP9*. Although not all of these genes ranked among the top cytoHubba candidates, their network connectivity and sequential functional relationships supported their separate retention as melanogenesis-associated candidates. Because the three categories differed in both network architecture and biological characteristics, candidate genes were classified using category-specific evidence. The line-background-associated category primarily retained microenvironment-related nodes, including *ADAMTS16*, *IL36B*, *EPHA6* and *CNTN6*; the region-associated category prioritized metabolism- and redox-related nodes, including *CAGE1*, *C1QL1*, *DGAT2L6* and *HAGHL*; and the shared category retained structural and cytoskeletal nodes, including *ATP2A1*, *RYR1*, *TRIM54*, *GMPR* and *ABRA*. The complete candidate-gene classification and supporting evidence are summarized in Figure 5F and were used to guide subsequent RT-qPCR validation.

### 3.6. Signature Scoring Reveals Sample-Level Variation in Skin-Associated Transcriptional States

**Figure 6:**
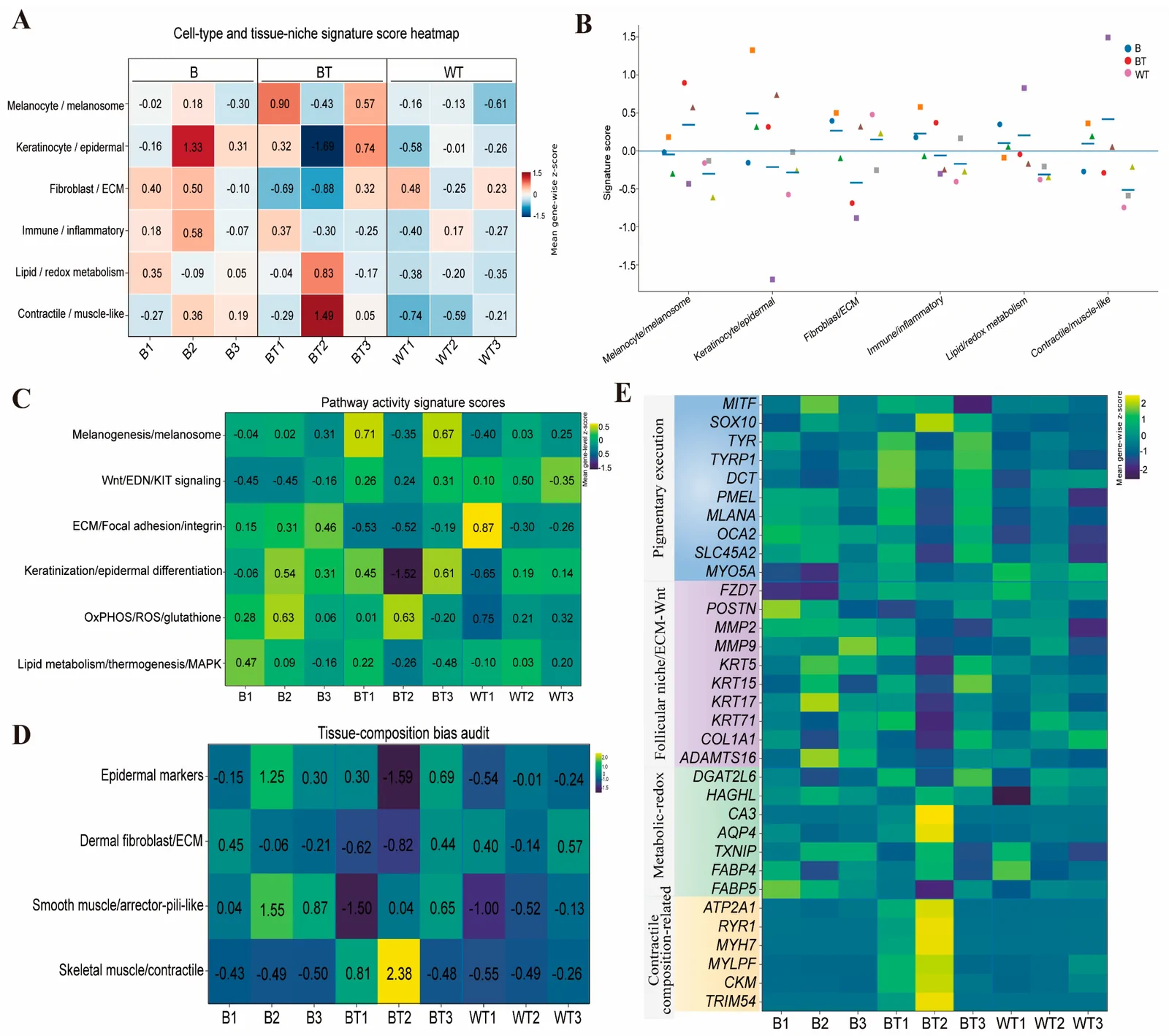
Signature scoring reveals sample-level variation in skin-associated transcriptional states. (**A**) Heatmap of marker-based cellular and tissue-associated signature scores across B, BT, and WT samples. (**B**) Distribution of the six marker-based signature scores across individual samples. Circles, squares, and triangles denote replicate labels 1, 2, and 3, respectively; point colors distinguish individual samples within the B, BT, and WT groups, and short horizontal lines indicate group means. Replicate labels are used only to distinguish biological replicates and do not indicate biological pairing across groups. (**C**) Heatmap of pigmentary and skin-associated signature patterns. (**D**) Contractile and tissue-composition-related signature patterns. (**E**) Expression heatmap of prioritized candidate genes.

To examine how the different transcriptional categories were distributed across individual samples, signature scoring was performed for the B, BT, and WT groups using predefined model gene sets (Figure 6). The signatures covered pigmentation-related programs, epidermal and extracellular matrix features, immune and inflammatory signals, metabolic and redox processes, and contractile tissue-associated components.

Pigmentation-related scores showed the clearest variation among samples. BT1 and BT3 exhibited relatively high scores for melanocytes, melanosome biogenesis, and melanogenesis, whereas WT samples generally showed lower scores; BT2 did not fully follow the pattern observed in BT1 and BT3 (Figure 6A–C). This distribution was broadly consistent with the expression heatmap of *MITF*, *SOX10*, *TYR*, *TYRP1*, *DCT*, *PMEL*, *MLANA*, *OCA2*, *SLC45A2* and *MYO5A*, which were generally more highly expressed in BT1 and BT3 (Figure 6E). In contrast, signatures related to Wnt, EDN, and KIT signaling, as well as keratinization, ECM adhesion, oxidative phosphorylation, redox regulation, and lipid metabolism, did not show clear and consistent separation among groups, but instead displayed stronger sample-dependent variation. The individual-sample dot plot likewise showed that some signatures had higher mean scores in the B or BT groups but also substantial within-group dispersion (Figure 6B).

Within the shared category, the presence of *ATP2A1*, *RYR1*, *MYH7*, *MYLPF* and *CKM* corresponded to elevated skeletal muscle and contractile-structure scores in selected BT samples (Figure 6D). Scores related to smooth muscle and arrector pili structures, the epidermis, dermal fibroblasts, and the ECM also varied among samples. These patterns raise the possibility that part of the shared transcriptional signal may be influenced by differences in tissue composition; however, this interpretation remains hypothetical because tissue composition and sampling depth were not independently assessed histologically.

### 3.7. Transcriptome-Based Hypothesis of Candidate Gene Signatures Associated with Coat-Colour Variation in Junken Meat Sheep Skin

**Figure 7:**
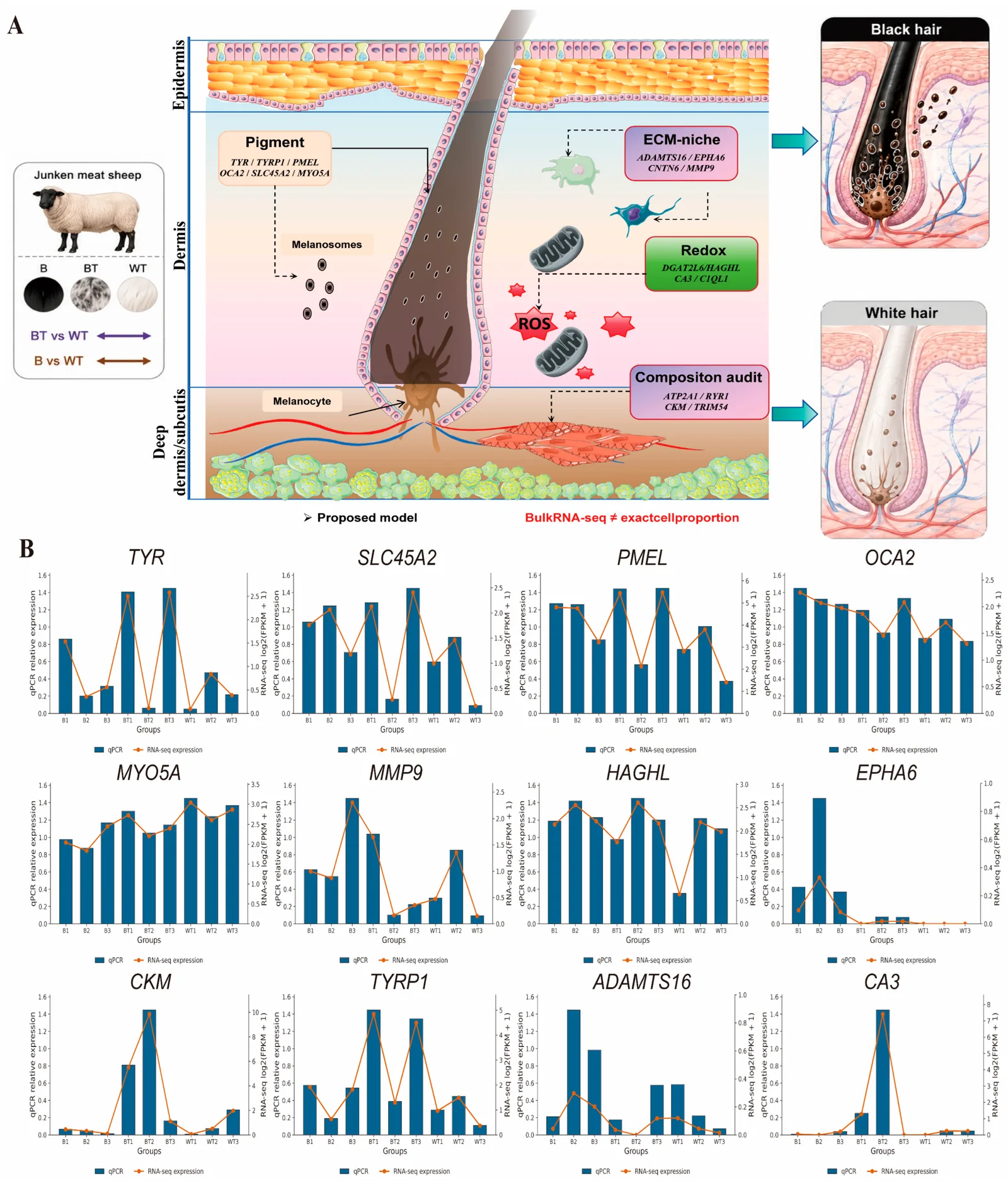
Transcriptome-derived candidate gene signatures associated with coat-colour variation in Junken meat sheep skin. (**A**) Schematic organization of candidate genes into pigmentary, ECM–niche, redox/metabolic, and composition-related categories. (**B**) Comparison of the expression patterns of 12 selected candidate genes determined by RT-qPCR and RNA-seq.

By integrating DEG stratification, functional enrichment, network-based prioritization, and signature scoring, the candidate genes were grouped into four categories: Pigment, ECM–niche, Redox/metabolic, and Composition audit, and were mapped onto a schematic representation of the skin–hair follicle unit (Figure 7A). The Pigment category included *TYR*, *TYRP1*, *PMEL*, *OCA2*, *SLC45A2* and *MYO5A*, which are mainly associated with melanin synthesis, melanosome maturation, and intracellular transport. *ADAMTS16*, *EPHA6*, *CNTN6* and *MMP9* were assigned to the ECM–niche category to summarize signals related to matrix remodeling, cell adhesion, and the perifollicular microenvironment. The Redox/metabolic category comprised *DGAT2L6*, *HAGHL*, *CA3* and *C1QL1*, reflecting regional differences in lipid metabolism, redox regulation, and local metabolic state. *ATP2A1*, *RYR1*, *CKM* and *TRIM54* were included in the Composition audit category as markers of expression signals potentially influenced by sampling depth, contractile tissues, or other aspects of tissue composition. This schematic was intended to position the candidate genes within four conceptual layers—pigmentary output, follicular microenvironment, metabolic state, and tissue composition—rather than to imply established causal regulatory relationships among them.

Twelve candidate genes were subsequently examined by RT–qPCR. Their overall expression patterns across the B, BT, and WT samples were broadly consistent with those observed by RNA-seq (Figure 7B).

## 4. Discussion

Rather than pooling all black- and white-haired skin samples into a single comparison, we interpreted the BT–WT and B–WT contrasts separately because they represent distinct but complementary biological contexts. Previous transcriptomic studies of sheep and goat skin have repeatedly identified differential expression of *TYR*, *TYRP1*, *PMEL* and genes involved in melanosome transport, demonstrating that RNA-seq can robustly capture coat-color-associated transcriptional signals [19,20,21]. However, the biological meaning of these signals depends strongly on the underlying comparison design. Because BT and WT samples were collected from black- and white-haired regions of the same white-bodied, black-headed individuals, this paired comparison reduces variation attributable to individual genetic background and systemic physiological status and therefore provides a useful within-individual view of regional coat-color-associated differences in skin and follicular state. Nevertheless, anatomical region and coat color differ simultaneously between BT and WT, and the resulting transcriptional differences should not be interpreted as pigmentation-specific effects alone. By contrast, B and WT samples were both obtained from dorsal trunk skin, thereby maintaining a comparable anatomical sampling site, but originated from different animals with distinct coat-color phenotypic backgrounds. Consequently, the B–WT comparison may capture coat-color-associated transcriptional variation together with between-animal and genetic or breeding-background differences, which may also reflect historical selection within the breeding population [22,23,24,25]. Accordingly, DEGs identified in B–WT should not be attributed solely to pigmentation, but rather interpreted as a composite transcriptional signature associated with phenotypic background and local skin state. Taken together, the two comparison frameworks provide complementary perspectives on coat-color-associated transcriptional variation while also defining the boundaries within which each contrast should be interpreted. This context-dependent interpretation is consistent with tissue-expression atlases and cross-tissue regulatory studies showing that tissue origin, genetic background, and the local microenvironment can all contribute substantially to transcriptomic variation [26,27,28].

Within these interpretive boundaries, the pigmentation-associated differences observed in black-haired skin can be placed in the established framework of mammalian melanogenesis. *TYR*, *TYRP1* and *DCT* represent major enzymatic components of melanin synthesis [29], whereas *PMEL* contributes to the fibrillar matrix of premelanosomes and provides a structural scaffold for subsequent melanin deposition [30,31]. The intraluminal environment of the melanosome is also critical for pigment production: *OCA2* contributes to anion conductance and organelle homeostasis [32], while *SLC45A2* modulates melanosomal pH and thereby influences melanocyte pigmentation capacity [33]. The positioning and transport of mature melanosomes further depend on the RAB27A–MLPH–MYO5A complex [34,35]. The coordinated expression of *TYR*, *TYRP1*, *DCT*, *PMEL*, *MLANA*, *OCA2*, *SLC45A2* and *MYO5A* across black- and white-haired skin is therefore more consistent with a continuous pigmentation-effector program encompassing melanin synthesis, melanosome formation and maturation, and intracellular trafficking than with the isolated action of any single gene. Similar expression patterns have been reported in multiple transcriptomic studies of sheep and goat skin [36,37], suggesting that this pigmentation axis may be partly conserved across small ruminants. RT–qPCR further supported the expression trends of *TYR*, *TYRP1*, *PMEL*, *OCA2*, *SLC45A2* and *MYO5A*. Nevertheless, the current evidence remains transcriptional, and confirmation of protein localization, melanin content, and functional relevance will require additional experimental validation.

Pigmentation is not an isolated process confined to melanocytes. The follicular pigmentary unit is embedded within a local environment composed of the epidermis, dermal papilla, extracellular matrix (ECM), and multiple neighboring cell populations. Hair follicle development and regeneration depend on sustained cell–ECM interactions, while the dermal papilla provides a regulatory niche for epithelial stem and progenitor cells and is itself shaped by ECM abundance and cell number [38,39,40]. In this context, the enrichment of ECM–receptor interaction, focal adhesion, integrin signaling, keratinization, and cytoskeletal processes in the shared category suggests that both comparisons involved alterations in the structural state of the epidermal–follicular unit and in cell–matrix adhesion. These results do not demonstrate that ECM signaling directly determines coat color; rather, they suggest that pigmentation differences are accompanied by changes in the perifollicular tissue environment. Previous studies have shown that loss of β1 integrin disrupts basement membrane formation, epidermal proliferation, and follicular downgrowth, while integrin-linked kinase is also required for epidermal and hair follicle morphogenesis [41,42]. The dynamic expression of tenascins, proteoglycans, and multiple adhesion molecules during follicle formation further reflects continuous remodeling of ECM–cell contacts [43]. Accordingly, the adhesion- and cytoskeleton-related signals in the shared category are more appropriately interpreted as part of the structural context of the skin–hair follicle unit rather than as an independent pigmentation-regulatory pathway. The line-background-associated candidates *ADAMTS16*, *IL36B*, *EPHA6* and *CNTN6* further point to matrix remodeling, inflammation-related signaling, and cell-contact regulation. Because direct evidence linking these genes to follicular pigmentation remains limited, they are better regarded as candidate markers of the skin microenvironment than as direct melanogenic factors. *MMP9* requires separate consideration: although it was connected to pigmentation-associated genes, including *TYR*, *TYRP1*, *PMEL*, *OCA2* and *SLC45A2* within the same subnetwork, its established functions are primarily related to ECM degradation and tissue turnover [44]. *MMP9* may therefore link pigmentation-associated transcriptional signals to the surrounding matrix state, but network proximity alone is insufficient to establish a direct role in melanin synthesis.

Epidermal signals provide an additional layer of support for this interpretation. After melanosome formation, pigment must be transferred to neighboring keratinocytes, meaning that the final distribution of pigment depends on coordinated interactions between melanocytes and keratinocytes rather than on melanocyte activity alone [45,46]. Single-cell transcriptomic studies in sheep likewise indicate that hair follicle morphogenesis involves multiple cellular states and locally organized microenvironmental signals [47]. Together with the sample-level heterogeneity observed in ECM-, epidermal-, and composition-related signature scores, the most cautious interpretation is that coat-color differences in Junken meat sheep are accompanied not only by changes in pigmentation-effector genes, but also by variation in perifollicular matrix organization, adhesion relationships, and epidermal state. However, the direction and magnitude of these changes were not fully consistent across all samples.

Beyond pigmentation-effector and follicular-structural signals, the region-associated category displayed a pronounced metabolic profile involving mitochondrial function, oxidative phosphorylation, reactive oxygen species (ROS)-related processes, thermogenesis, and ether lipid metabolism. Melanin synthesis imposes an oxidative burden on melanocytes, while melanocyte survival and pigment output are themselves sensitive to oxidative stress, supporting a close relationship between the local redox environment and melanogenesis [48]. The thioredoxin and glutathione systems help maintain intracellular redox balance and can modulate melanogenesis-related reactions [49]. The accumulation of H_2_O_2_ and impaired methionine sulfoxide repair observed in human hair graying further illustrate the sensitivity of the follicular pigmentary system to sustained oxidative damage [50]. However, regional coat-color differences in Junken meat sheep are not equivalent to age-related hair graying; these studies therefore support only the possibility that redox state influences follicular pigmentation and do not establish elevated ROS as a direct cause of regional coat-color variation. Mitochondrial and energy-metabolic signals may also reflect the broader physiological state of the hair follicle. Hair follicle stem-cell differentiation is accompanied by increased aerobic respiration; mitochondrial dysfunction can delay hair growth; and lactate metabolism contributes to hair follicle stem-cell activation [51,52]. Accordingly, the oxidative phosphorylation and thermogenesis signals detected in the region-associated category may more plausibly reflect differences in energetic demand, cellular activity, or local tissue state between black- and white-haired regions. MAPK signaling was also enriched in the region-associated profile and may be relevant to pigmentation-associated signaling. However, the present bulk RNA-seq data do not establish a mechanistic link between metabolic stress, MAPK activity, and pigmentation. Classical studies have shown that MAPK can transmit upstream signals such as KIT to MITF and thereby influence melanocyte transcriptional programs [53]; however, in bulk RNA-seq data, MAPK-related signals may also originate from keratinocytes, fibroblasts, or other skin cell populations and cannot be equated directly with MITF activation within melanocytes. The region-associated candidates *HAGHL*, *CA3*, *DGAT2L6* and *C1QL1* should likewise be interpreted at different functional levels: *CA3* is more closely related to local acid–base buffering and metabolic state, *DGAT2L6* suggests differences in lipid synthesis or remodeling, whereas *HAGHL* and *C1QL1* are currently retained mainly as candidate nodes associated with redox balance or the local signaling environment. Ether lipids and plasmalogens contribute to membrane integrity and protection against oxidative damage [54], whereas members of the *DGAT* and *AWAT* families participate in neutral-lipid and wax-ester metabolism [55]. These functional contexts provide plausible explanations for the lipid-metabolism enrichment but remain insufficient to define the specific role of *DGAT2L6*. Overall, the region-associated category appears to capture differences in metabolic and redox states across skin regions rather than a firmly established pathway in which metabolism directly drives coat-color variation.

Unlike single-cell or spatial omics, bulk RNA-seq captures the averaged transcriptional signals of multiple cell populations and microanatomical structures within the sampled tissue. Consequently, some differentially expressed genes or enriched categories may not necessarily represent regulatory changes occurring within the same cell type, but may instead arise from differences in cell proportions, tissue layers, or sampling depth. CIBERSORT, GSVA, xCell, and subsequent benchmarking studies of transcriptomic deconvolution have all emphasized that bulk expression profiles can be used to infer cellular composition or pathway activity; however, such inferences depend on the reference signatures, gene-set definitions, and sample composition and therefore cannot be directly equated with cell-level mechanisms [56,57,58,59]. In the present dataset, muscle-contraction- and myofiber-related genes, including *ATP2A1*, *RYR1*, *CKM*, *TRIM54*, *MYH7* and *MYLPF*, showed prominent transcriptional signals. These patterns may partly reflect differences in deep-dermal, arrector-pili-associated, or other contractile tissue components captured during bulk skin sampling. However, because sampling depth and tissue composition were not assessed histologically, this interpretation remains hypothetical.

The hair follicle is not an isolated pigment-producing unit, but is embedded within a complex microenvironment comprising the epidermis, dermis, follicular mesenchyme, vasculature, immune cells, adipose tissue, and arrector pili-associated structures. Previous studies have shown that the basement membrane of hair follicle stem cells can function as a niche for muscle cells, indicating close anatomical and molecular interactions between hair follicles and adjacent contractile structures [60]. Single-cell transcriptomic analyses have further revealed pronounced differentiation hierarchies and spatial features among epidermal and follicular cell states [61], while both the cellular composition and transcriptional landscape of the skin change systematically between hair growth and resting phases [62]. The muscle-like, structural, and deep-tissue-associated signatures observed in the present study may therefore represent composition effects captured by bulk sampling of a complex skin tissue. Spatial transcriptomics preserves the anatomical localization of gene expression within tissue sections and thus provides a more direct approach for distinguishing genuine intracellular regulation from local differences in tissue composition [63]. Our previous transcriptomic study of coat-color transition similarly showed that pigmentation changes involve not only *TYR*, *TYRP1*, *PMEL*, *OCA2* and *SLC45A2*, but also expression changes related to the ECM, follicular microenvironment, and metabolism, thereby supporting a broader “follicular microenvironment–melanogenesis axis” framework [64].

In summary, based on bulk RNA-seq data, we propose a working model for interpreting coat-color-associated differences in Junken meat sheep skin, comprising three candidate biological programs together with a tissue-composition audit. The Pigment layer encompasses melanin synthesis, melanosome maturation, and pigment transport; the ECM–niche layer reflects the state of the perifollicular matrix, cell adhesion, and local microenvironment; and the Redox/metabolic layer points to potential differences in metabolic and redox states among skin regions. The Composition audit further indicates that some contractile or muscle-like signals may arise from variation in dermal depth, arrector pili-associated structures, or sampling depth rather than from regulatory changes within a single cell type. Previous studies have established *ASIP* and *MC1R* as major genetic determinants of coat color in sheep [65,66,67,68]. However, because the present study did not assess genomic variation at these or other pigmentation-associated loci, our findings should be regarded primarily as complementary evidence at the transcriptional level. Collectively, the coat-color-associated differences observed in Junken meat sheep are best represented as a multilayered transcriptional landscape involving pigmentation-effector programs, the follicular microenvironment, metabolic and redox states, and tissue composition. This framework provides testable candidates for subsequent histological localization and functional validation.

## 5. Conclusions

By integrating bulk RNA-seq data from distinct coat-color lines and skin regions of Junken meat sheep, this study distinguished region-associated, line-background-associated, and shared transcriptional modules. Differences between black- and white-haired skin involved not only a pigmentation-effector program comprising *TYR*, *TYRP1*, *PMEL*, *OCA2*, *SLC45A2* and *MYO5A*, but also changes in extracellular matrix remodeling, cell adhesion, epidermal keratinization, mitochondrial metabolism, and redox state. Some contractile and muscle-like signals may reflect variation in dermal depth, arrector pili-associated structures, or sampling depth. On this basis, we propose a multilayered candidate framework comprising Pigment, ECM–niche, Redox/metabolic, and Composition audit components. Future integration of histological analysis, spatial or single-cell transcriptomics, protein localization, and functional assays will be required to resolve the cellular origins of these transcriptional programs and clarify their contributions to coat-color formation in Junken meat sheep.

## Data Availability

The RNA-seq datasets generated and analysed during the current study are available in the NCBI Sequence Read Archive repository under BioProject accession number PRJNA1492038.

## Supplementary Materials

The following supporting information can be downloaded at: https://www.mdpi.com/article/10.3390/biology15171457/s1, Table S1: Sample information and experimental comparison design; Table S2: Primer information used for RT-qPCR; Table S3: Summary of RNA-seq data output and basic sequencing quality in BT, WT and B skin samples; Table S4. Marker gene sets and sample-level scores used for signature analysis.
