# Supplementary material for: Comparative Transcriptomic Profiling Identifies Region- and Phenotypic-Background-Associated Transcriptional Programs Linked to Coat-Color Variation in Junken Meat Sheep
Source: Biology (Basel). 2026 Aug 26;15(17):1457. doi: 10.3390/biology15171457 (PMC13565083; doi:10.3390/biology15171457)
Supplement: Supplementary file 1 [file biology-15-01457-s001.zip › Supplementar Tables S1-S3.pdf]

Supplementary Table S1. Sample information and experimental comparison design.

Individual ID	Sample ID	Sex	Age/developmental stage	Coat-color status at sampling	Sampling site
Individual 1	BT1	Male	M1	black-haired head skin	Standardized dorsal trunk skin
Individual 1	BT2	Male	M1	black-haired head skin	Standardized dorsal trunk skin
Individual 1	BT3	Male	M1	black-haired head skin	Standardized dorsal trunk skin
Individual 2	WT1	Male	M1	white-haired dorsal trunk skin	Standardized dorsal trunk skin
Individual 2	WT2	Male	M1	white-haired dorsal trunk skin	Standardized dorsal trunk skin
Individual 2	WT3	Male	M1	white-haired dorsal trunk skin	Standardized dorsal trunk skin
Individual 3	B1	Male	M1	black-haired dorsal trunk skin from lambs born entirely black	Standardized head skin
Individual 3	B2	Male	M1	black-haired dorsal trunk skin from lambs born entirely black	Standardized head skin
Individual 3	B3	Male	M1	black-haired dorsal trunk skin from lambs born entirely black	Standardized head skin

Supplementary Table S2. Primer information used for RT-qPCR.

Gene Name	Primer Sequences (5' - 3')	Product (bp)
MYO5A	F: GATGGAGAGCTGTTGCTGGT<br>R: GGCTCCTCTTCTGAGACTGC	85
TYR	F: ACAACAGTCGCCAGGCTTTA<br>R: TCCTGGCTTTGTCGTGGTTT	81
TYRP1	F: AACACCTGCGACATTTGCAC<br>R: CGCAGACTACTCGCCATTGA	106
PMEL	F: CAGAAGGTACTGCAGGCTCC<br>R: GTCCAGGGTGAGGGAAAAGG	97
OCA2	F: CGTGCTCCTAAACCTGAGCA<br>R: GTTACCTCCCAGACAGGCAC	94
MMP9	F: CCCGGATCAAGGATACAGCC<br>R: TGGCACGGAGGAGTGATCTA	82
HAGHL	F: ACTGACCCCTCCCGCAC<br>R: ATGACCTGCAGAGTTCAGCC	103
SLC45A2	F: CCCAACCGGAGACCCTACATCCTCA<br>R: GATATGATCGCGTCCCCGTT	117
EPHA6	F: ATGGGTCAGTTTGACCATCCA<br>R: GCAGAACGTTTCCACCCCAA	90
CKM	F: GTCGAGCTGTCTCTGGAAGG<br>R: GTGCTTGTTGTGGGTGTTACC	75
CA3	F: AACTGCTCTGAAGCATCCCG<br>R: CGCCCTTCTCACGTCCTATC	71
DAMTS16	F: GCACCATCATGAAGCACGAC<br>R: AGAAGTAGCAGGCCAGAACG	120

GADPH	F: CTGACCTGCCGCCTGGAGAAA<br>R: GTAGAAGAGTGAGTGTCGCTGTT	180

Supplementary Table S3. Summary of RNA-seq data output and basic sequencing quality in BT, WT and B skin samples.

	Type	BT	WT	B
RNA-seq	Raw reads (M)	42.38±2.14	44.29±0.52	45.29±2.49
	Raw bases (G)	6.36±0.32	6.64±0.08	6.79±0.38
	Clean reads (M)	41.09±2.63	43.11±0.71	44.30±2.42
	Clean bases (G)	6.17±0.40	6.47±0.11	6.65±0.36
	Error rate (%)	0.02±0.00	0.02±0.01	0.03±0.00
